# Tofacitinib Citrate for Ulcerative Keratitis in a Patient with Rheumatoid Arthritis

**DOI:** 10.1155/2014/403452

**Published:** 2014-06-17

**Authors:** Philip B. Meadow, Jacqueline Nguyen, Keerthana Kesavarapu

**Affiliations:** ^1^Rivertown Rheumatology P.C., Columbus Regional Health, Columbus, GA 31901, USA; ^2^Kirksville College of Osteopathic Medicine, Kirksville, MO 63501, USA; ^3^Warren General Hospital, Warren, PA 16365, USA; ^4^Western Reserve Health Care System, Youngstown, OH 44501, USA; ^5^Wilford Hall Medical Center, Lackland Air Force Base, San Antonio, TX 78236, USA; ^6^Philadelphia College of Osteopathic Medicine, Suwanee, GA 30024, USA

## Abstract

*Purpose.* To report a case of a patient with rheumatoid arthritis (RA) treated with tofacitinib citrate.* Methods.* Observational case report.* Results.* A 59-year-old patient, with a history of rheumatoid arthritis, on methotrexate 10 mg PO qwk and IV abatacept 750 mg/month, presented with photosensitivity, foreign body sensation, pain, redness, and blurry vision of her right eye (RE). Visual acuity of the RE was 20/200 and 20/20 of the left eye (LE). The slit lamp examination of the RE revealed dryness, 2+ injection of the conjunctiva, and pericentral ulceration of the cornea with 20–30% stromal thinning, pannus, and diffuse punctate epithelial erosions. The anterior chamber appeared normal. Laboratory values revealed elevated levels of rheumatoid factor, anticyclic citrullinated peptide antibodies, and C-reactive protein. The patient was switched to tofacitinib citrate 5 mg PO b.i.d, underwent corneal gluing, and was given prednisone acetate 1% gt TID, polytrim gt TID, neomycin-polymyxin-dexameth gt QD, FreshKote lubricant 1.8% gt QID, moxifloxacin 0.5% gt QID, and preservative free artificial tears Q1H. Within one week, laboratory values normalized, symptoms diminished, and the cornea reepithelialized.* Conclusion.* RA can present with ulcerative keratitis. Tofacitinib citrate, steroids, and corneal gluing were found to halt the progression of keratolysis and promote reepithelialization.

## 1. Background

Rheumatoid arthritis (RA) is a chronic inflammatory disease which primarily afflicts joints but can manifest as extra-articular symptoms. Often, RA patients present with ocular complications in their clinical course, including keratoconjunctivitis sicca, uveitis, corneal impairment, scleritis, and ulcerative keratitis (UK) [1]. UK, or corneal ulceration, is a rare and late complication of RA and can progress to a corneal perforation and become an ocular emergency [2]. While these ulcerations can materialize either centrally or peripherally, they tend to develop more commonly in the periphery. The peripheral cornea is well-vascularized with increased access to inflammatory cells compared to the avascular central cornea. As a result, patients will commonly present with a painful red eye and can less commonly have an excessive watery eye, a feeling of foreign body in the eye, or reduced visual acuity [3].

One favored hypothesis of mechanism for corneal ulceration in patients with RA stems from an abnormal B and T cell interaction and increased cytokine production, specifically tumor necrosis factor (TNF) and interleukin-6 (IL-6), seen in autoimmune disease [4]. Elevation of expression of these cytokines leads to an imbalance between collagenases, specifically matrix metalloproteinases (MMPs), and tissue inhibitors, specifically tissue inhibitor of metalloproteinases-1 (TIMP-1). This imbalance leads to a build-up of collagenases in the cornea allowing for destructive keratolysis [3]. Smith et al. suggested that MMP-2, which is produced in the corneal stroma, and MMP-9, which is produced in the lacrimal glands, [5] lead to corneal thinning, corneal ulceration, and dry eye syndrome.

Currently, the recommended systemic medical management for RA patients with ocular complications is NSAIDS, oral corticosteroids, and systemic immunosuppressive chemotherapy. Traditional first-line therapy for RA-associated ulcerative keratitis is systemic corticosteroids; however, they are often unable to halt disease progression. If the corneal ulcerations are nonresponsive to corticosteroids, aggressive immunosuppression is indicated using combination of cyclophosphamide, methotrexate, azathioprine, and cyclosporine [6]. One study presented three cases with resolution of RA-associated ulcerative keratitis with the use of infliximab, a disease modifying antirheumatic drug (DMARD). The pathogenesis suggests that inhibiting TNF-alpha allows for a decrease in MMPs,which would reduce the risk of corneal stroma degradation [2, 6, 7]. Similar findings and mechanisms were involved in the use of other TNF*α* inhibitors: etanercept, adalimumab, and rituximab, [8, 9].

Another DMARD tofacitinib is reserved for patients with moderate-to-severe active RA with an inadequate response or intolerance to previous DMARD therapy. Tofacitinib citrate inhibits the Janus kinase (JAK) pathway which is critical for immune cell activation, proinflammatory cytokine production, and cytokine signaling [10, 11]. The immunomodulatory effects allow the drug to reduce and alleviate the inflammatory processes leading to and sustaining articular changes as well as corneal ulcerations [11]. Specifically, the JAK pathway decreases levels of MMPs and IL-6, which are upregulated on corneal epithelial cells in response to injury or inflammation [10, 12]. While it is effective in resolving symptoms of RA, no previous study or case report has documented its role in allowing for reepithelialization of the cornea and therefore improving symptoms of corneal ulcerations. The aim of this study is to report a case of an RA patient with associated peripheral UK novelly treated by tofacitinib citrate.

## 2. Case Report

A 59-year-old female patient with a 9-year history of rheumatoid arthritis (RA), under immunosuppressive therapy (methotrexate 10 mg PO qwk and IV abatacept 750 mg/month), presented with photosensitivity, foreign body sensation, pain, redness, and blurry vision of her right eye (RE) for one month in 2013. On examination, visual acuity of the RE was 20/200 and 20/20 of the left eye (LE). The slit lamp examination of the RE revealed dryness, 2+ injection of the conjunctiva, and pericentral ulceration of the cornea with 20–30% stromal thinning, pannus, and diffuse punctate epithelial erosions (PEE). The anterior chamber appeared normal, deep and quiet. The lesions were classified as sterile corneal keratitis. The nonhealing ulcerations were likely due to underlying dry eye and RA flare-ups with no signs of infectious etiology.

Laboratory investigations of RA revealed elevated levels of rheumatoid factor (RA latex turbid: 842.0 IU/mL, normal range: 0–20 IU/mL), anticyclic citrullinated peptide antibodies (anti-CCP level: 192 IU/mL, normal range: <30 IU/mL), and C-reactive protein (CRP level: 98.2 IU/mL, normal range: 0–0.8 IU/mL). To monitor disease activity, the rheumatologist closely followed CRP levels which strongly correlate with inflammation and predicts RA severity. For several years, CRP levels mostly remained low (fluctuating between <0.5 and 4.5 IU/mL) with occasional elevations during RA flare-ups (up to 8.2 IU/mL) approximately once per year. Beginning in 2011, our patient experienced more frequent RA flare-ups with CRP levels regularly increasing to 34.3 IU/mL and finally to 98.2 IU/mL. The dosage of IV abatacept was increased from 500 mg/month to 750 mg/month which provided temporary relief.

With the onset of corneal changes, the patient was admitted to the hospital under observation and was diagnosed with corneal melting syndrome. She was treated with methylprednisolone sodium succinate in the hospital and was discharged to follow up with West Georgia Eye Care Center. Ophthalmic medications included prednisone acetate 1% 1 drop TID, polytrim 1 drop TID, neomycin-polymyxin-dexameth 1 drop QD, FreshKote lubricant 1.8% 1 drop QID, moxifloxacin 0.5% 1 drop QID, and preservative free (PF) artificial tears Q1H. The patient also underwent corneal gluing procedure of the RE. the rheumatologist replaced IV abatacept with tofacitinib citrate 5 mg PO b.i.d. Joint pain significantly decreased, CRP levels dropped to 1.3 IU/mL, and reepithelization of the cornea occurred within 1 week of initiation of new immunosuppressive therapy and corneal gluing procedure.

On follow-up visits, our patient reported no vision complaints, eye pain, or discomfort. The visual acuity of the RE improved to 20/40 in the first 2 weeks and 20/30 within 1 month. Slit lamp examination revealed stromal scar and cornea and no longer thinning. The patient was instructed to use prednisone acetate 1% 1 drop b.i.d and artificial tears (PF) 1 drop p.r.n. The patient's RA appeared to be in near remission with diminished sensation of pain, stiffness, and swelling in her joints.

## 3. Discussion 

In the presented case, the patient had high levels of CCP and RA turbid factor which are associated with worse clinical outcomes and extra-articular manifestations in rheumatoid arthritis. The patient presented with keratoconjunctivitis sicca, corneal ulcerations, and a corneal perforation, all of which responded well to a combination therapy of tofacitinib citrate, methotrexate, and corneal glue. This combination aided in halting the keratolysis and encouraged reepithelialization. Because of the serious complications of peripheral ulcerations of the cornea, it is vital that patients be closely monitored and open communication is established between the ophthalmologist and the rheumatologist to avoid associated mortality and to control the underlying disease.

In the literature published to date, ophthalmic tofacitinib citrate has been shown to be an effective treatment for patients with dry eye disease (DED) with a good safety profile [11]; however, its use in corneal perforations has yet to be elucidated. Tofacitinib citrate has a novel mechanism as a JAK pathway inhibitor allowing a decrease in the inflammatory cells that are involved in the dissolution of the corneal stroma. Moreover, this drug works to decrease the imbalance between MMPs and TIMPS that is seen in patients with chronic RA which lead to corneal changes [10, 11]. Robust clinical trials and further pharmacoeconomic studies are necessary to position tofacitinib citrate amongst other DMARDs. In the meantime, it remains a mainstay of treatment for moderate-to-severe RA that is refractory to previous DMARD therapy [10].
